# Can we routinely identify the external branch of the superior laryngeal nerves with neural monitoring?: a prospective report on 176 consecutive nerves at risk

**DOI:** 10.1007/s13304-021-01084-6

**Published:** 2021-05-26

**Authors:** Paolo Del Rio, Elena Bonati, Tommaso Loderer, Matteo Rossini, Federico Cozzani

**Affiliations:** Department of Medicine and Surgery, General Surgery Unit, Parma University Hospital, Parma, Italy

**Keywords:** Cernea classification, The external branch of the superior laryngeal nerve, Intraoperative nerve monitoring, Thyroidectomy, Miniinvasive thyroidectomy

## Abstract

The external branch of the superior laryngeal nerve (EBSLN) provides motor function to the cricothyroid muscle (CTM). EBSLN damage produces changes in voice quality and projection. Intraoperative neuromonitoring (IONM) in thyroid surgery aims to optimize EBSLN control during dissection. We prospectively collected the data of 88 consecutive patients who underwent total thyroidectomy with IONM from July 2019 to December 2019. IONM was offered in the intermittent mode of application. We routinely searched for the EBSLN electromyographic (EMG) signal before (S1) and after (S2) dissection of the superior vascular peduncle. In the absence of the EMG signal, we observed the CTM twitch. We identified 141 (80%) S1 EMG signals, while we recorded the CTM twitch in 15 cases (8.5%). In 20 (11.3%) cases, we were unable to identify the EMG signal. Analysing the S2 results, we found loss of EBSLN signal in 11/141 cases (7.8%) identified with IONM in pre-dissection stimulation. Among the 20 cases without pre-dissection identification (we had not identified the external branch of the superior laryngeal nerve or the muscle twitch), in the post-dissection evaluation, we confirmed the loss of signal in 17 of 20 cases, equal to 85% (*p* < 0.001). Our data clearly show that intraoperative stimulation and recognition of EBSLN, performed before any dissection manoeuvre to the superior vascular thyroid pole, leads to a much higher rate of nerve conservation.

## Introduction

**Fig. 1:**
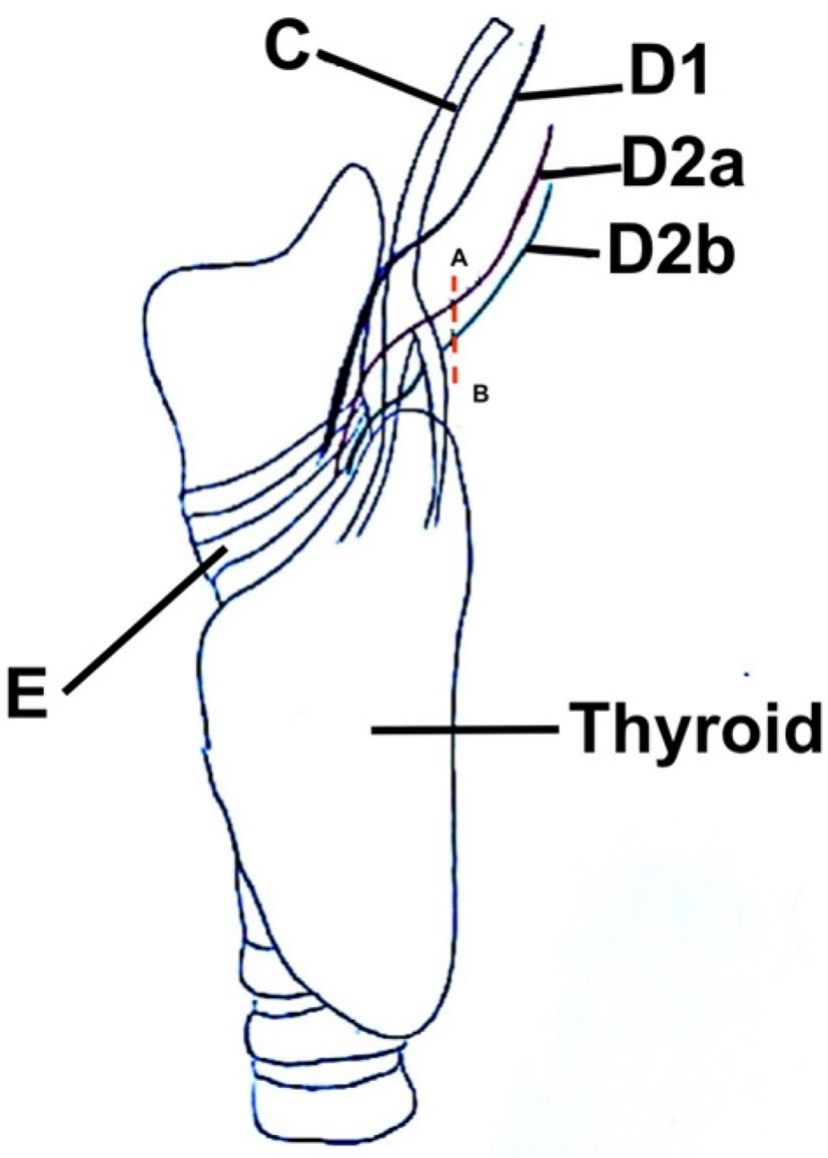
Cernea’s Classification. **a**–**b** = 1 cm. **c** = Superior Thyroid vessels. **d** = External Branch of the Superior Laryngeal Nerve. D1 = Type 1 Cernea. D2a = Type 2a Cernea. D2b = Type 2b Cernea. **e** = Crico Thyroidal muscle

The external branch of the superior laryngeal nerve (EBSLN) provides motor function to the cricothyroid muscle (CTM) [1]. There are few anatomical classifications of the EBSLN; the most widely adopted is the one proposed in 1992 by Claudio R. Cernea [2, 3]. This classification (Fig. 1) is based on the potential risk of nerve injury during thyroid surgery: the EBSLN is surgically relevant because it is in close anatomical proximity to the superior thyroid vessels (STVs), inferior constrictor muscle, CTM and thyroid cartilage.

EBSLN damage produces changes in voice projection and quality, alongside alterations in high-pitched sound production ability, altered frequency of voice, modified voice timbre, deteriorated voice performance, reduced voice quality projection, and increased effort to speak. EBSLN dysfunction-related symptoms may, in some cases, deeply and negatively influence the standard quality of life. Of note, these symptoms may be more noticeable with professional speakers (especially true in women).

EBSLN injury rate is reported to be up to 58% [4, 5].

However, the diagnosis of EBSLN dysfunction is difficult to confirm based solely on clinical or endoscopic findings [5].

Present and future research on intraoperative neural monitoring (IONM) in thyroid surgery aims to optimize EBSLN control during dissection and to close the interval between IONM results and pre- and postoperative assessments of vocal cord (VC) movement and voice quality.

In this clinical prospective study, IONM was used to assess the value of EBSLN identification, prognostication and stratification into the Cernea classification.

## Materials and methods

### Patients’ recruitment

We prospectively collected the data of 88 patients treated with total thyroidectomy using IONM from July 2019 to December 2019. Surgery was performed by surgeons with extensive experience in nerve monitoring. The inclusion criteria were patients undergoing thyroidectomy with standardized IONM and age of at least 18 years. Patients were not eligible for the study in cases of preoperative injury to the laryngeal nerves, previous neck surgery, lobectomies, surgery with only lymph node dissection, intraoperative findings of tumour involvement of laryngeal nerves or local anaesthesia. Data from these patients were removed from this analysis.

### Setting

Academic Division, Unit of General Surgical Clinic of the University Hospital of Parma.

### Study design

Prospective observational study.

### Ethics

The protocol was approved by the Institutional Review Board (prot.n.38133/2018 of University Hospital of Parma). Patients signed an informed consent form before surgery. The use of IONM was explained as an effort to improve laryngeal nerve identification.

### IONM standards

IONM was offered in the intermittent mode of application. Nerve monitoring was performed according to standards of equipment setup, induction and maintenance anaesthesia, correct tube positioning verification tests, and EMG definitions.

The standard steps of RLN and EBSLN monitoring in intraoperative nerve monitoring are as follows:

- L1: vocal cord examination with preoperative laryngoscopy
- V1: stimulation of the ipsilateral vagus before RLN dissection
- R1: stimulation of the RLN at the first point where it is found in the tracheoesophageal groove
- S1: stimulation of the EBSLN with a probe after it has been detected
- S2: stimulation of the EBSLN proximal to the point where superior thyroid vessels are separated, following separation of the vessels and successful bleeding control
- R2: stimulation of the RLN at its most proximal point after the dissection is complete
- V2: vagus stimulation after bleeding control is complete at the surgical field
- L2: vocal cord examination with postoperative laryngoscopy. ^(6)^

L1 was assessed in all patients a few weeks before surgery. L2 was assessed with vocal cord stroboscopy in patients with RLN signal loss six months after surgery.

### EBSLN monitoring and definitions

EBSLNs were stimulated using a single use, incrementing process stimulating probe, monopolar, standard flexible tip (product n.8225490, Medtronic, Jacksonville, Florida, USA), 100 ms impulse duration and 4 Hz frequency. An event threshold was set with a reduced response threshold to identify small responses at 50 μV. For visually identified EBSLNs, a 1 mA current was preferred, while for EBSLN mapping and dissection, a higher value was used (2–3 mA) [7].

We considered the following prerequisites for definition of loss of EMG EBSLN signal:

Vocal cord examination with preoperative laryngoscopy (L1): normal VC movement.

S1: initial EBSLN satisfactory EMG signal with/without CTM twitch.

S2: no EMG response.

**Fig. 2:**
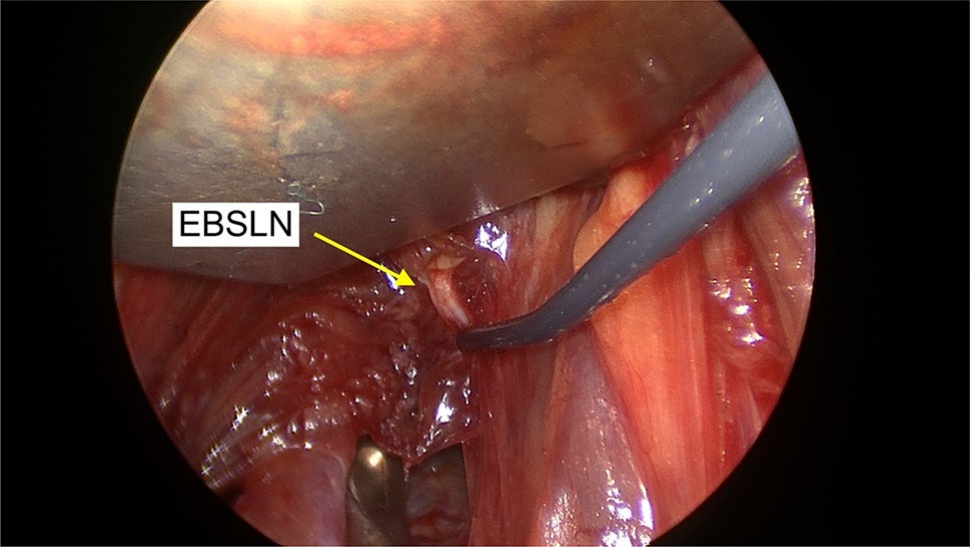
Post after STV ligation (S2)

In the current study, the EBSLN was identified and monitored using the following scheme:

(A) Dissection. The anatomy was defined and, in particular, the sternothyroid-laryngeal-superior pole triangle was identified. Gentle traction of the superior thyroid lobe was performed in the lateral and caudal directions to obtain good exposure of the sternothyroid-laryngeal triangle and the pars recta and pars obliqua of CTM. Meticulous dissection of the nonperfused area between CTM and the upper pole of thyroid was performed. To preserve the CTM and EBSLN, an accurate surgical technique was mandatory, with clear operational control and exposure of the sternothyroid-laryngeal triangle. Transverse division of the cranial portion of the sternothyroid muscle was performed with caution due to the possible proximity of the EBSLN.

(B) Mapping. The superior pole lateral position was maintained throughout all EBSLN identification, confirmation, and monitoring steps. Simultaneously, mapping the EBSLN was performed with a 1.5—2.0 mA current in the sternothyroid-laryngeal triangle, looking for a nerve structure, positive CTM twitch and EMG response.

(C) Nerve confirmation. Visual (nerve and CTM twitch) and electrical identification (clear biphasic waveform with recognizable amplitude) of the EBSLN was obtained before individual ligation of STV branches. It was important to test the most cranial portion of the nerve, stimulating the segment above the region of the nerve dissected during superior pole management so that the nerve could be adequately tested during this manoeuvre. The EBSLN EMG signal (S1) was obtained using a 1.0 mA current. For nerves located deep to the fascia of the inferior constrictor muscle, the EBSLN was located and mapped at 2.0 mA instead of by intramuscular dissection.

(D) Functional evaluation and classification. After complete STV ligation, thyroidectomy and haemostasis, the cranial aspect of the exposed EBSLN was stimulated with 1.0 mA (S2) (Fig. 2).

Anatomic variants of EBSLN were classified and described at surgery by a joint judgement between the first surgeon and the assistants.

### Outcomes

For each patient, we recorded the demographics, procedure, definitive pathology and whether EBSLNs were identified. We also recorded the Cernea classifications, EMG S1 and S2 findings and presence or absence of CTM twitch.

### Statistical analysis

All patient data were collected in a prospective manner with a dedicated electronic Microsoft Office Access Data Base (Microsoft Corp, Redmond, Wash). Unless otherwise stated, all data are expressed as the median and range. Statistical analysis was computed with SPSS, release 20.0 for Windows (SPSS Inc, Chicago-Ill, USA). The level of significance was set at *P* < 0.05.

## Results

### Patient characteristics, operative outcomes and pathology

**Fig. 3:**
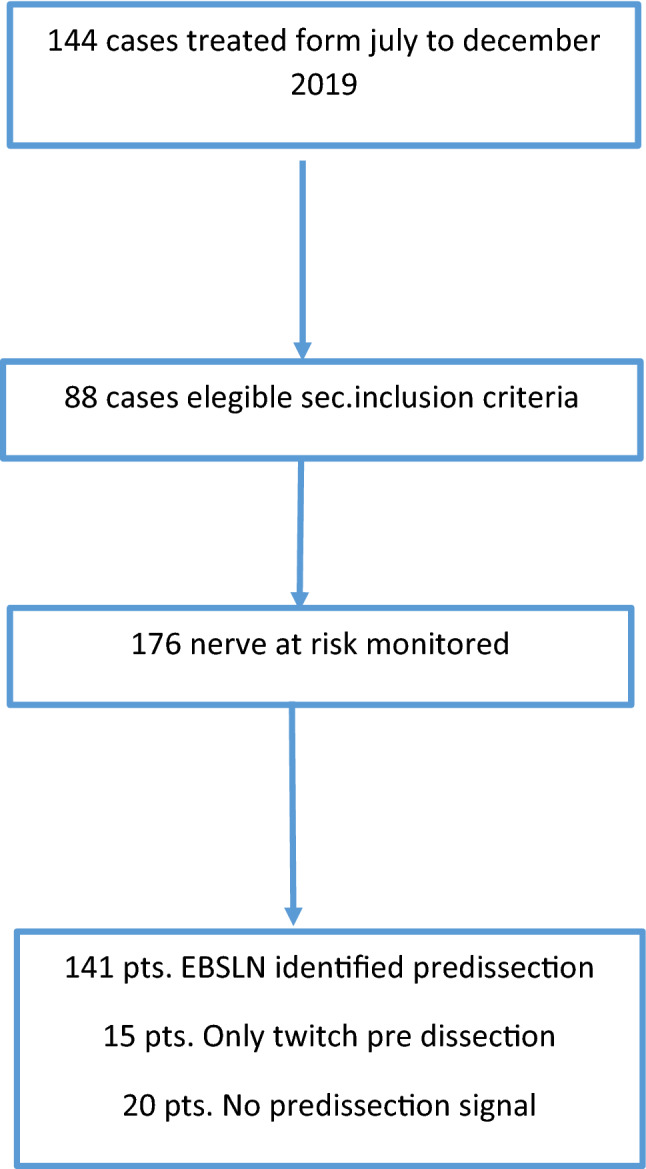
Analysis of cases

**Fig. 4:**
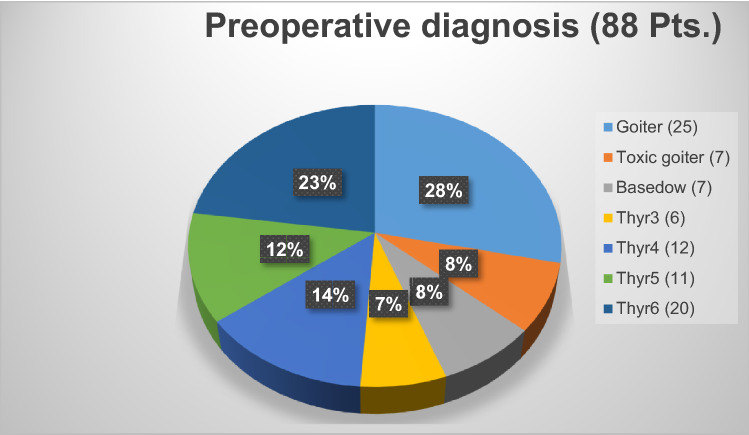
Preoperative diagnosis

We collected data on 176 potential nerves at risk (NAR) from 88 patients, considering both the inferior laryngeal nerve and the external branch of the superior laryngeal; all patients underwent total thyroidectomy according to the inclusion criteria, 18 with minimally invasive video-assisted technique (MIVAT) [8, 9] (Fig. 3). Our study included 64 female patients with a mean age of 51.22 ± 16.11 years and 24 male patients with a mean age of 52.33 ± 12.05 years (*p* = 0.76). Patients had a preoperative diagnosis of goitre in 25 cases, toxic goitre in 7 cases, Graves' disease in 7 cases, and Bethesda class 3 compatible cytology in 6 cases, class 4 in 12 cases, class 5 in 11 cases and class 6 in 20 cases. (Fig. 4).

The mean size of the nodules subjected to cytology was 15.5 mm (range 8–45 mm). The anatomical localization of the suspected nodules was found in the right lobe in 25 cases, in the left lobe in 14 cases, in the isthmus in 3 cases and bilateral in 7 cases. We lost the RLN signal (LOS) in two cases, but we did not perform two-stage thyroidectomy because both patients had bilateral localizations. Both signal losses were temporary and were resolved at a 6-month L2 control after a speech therapy cycle. In 7 out of 88 cases, we recorded transient postoperative paraesthesia that resolved within the first 7 days after the surgical procedure.

The mean operative time of the surgical procedures was 61.5 ± 1.5 min.

### EBSLN outcomes

**Fig. 5:**
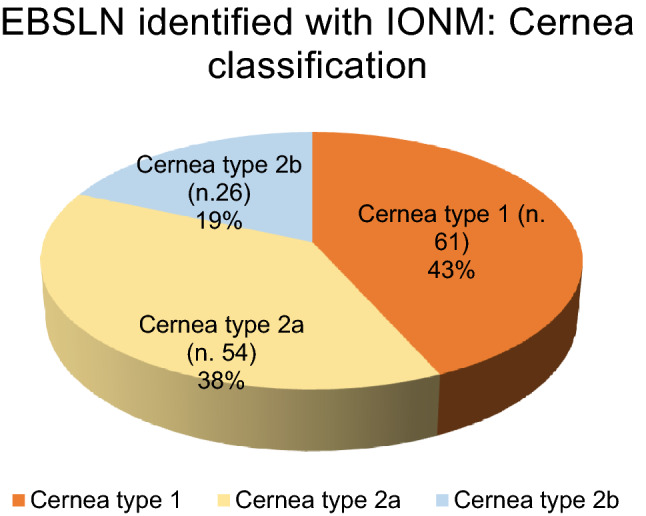
EBSLN identified with IONM: Cernea Classification

The identification of the S1 signal was carried out in all patients examined. We identified 141 S1 signals, equal to 80.1% of cases, while we recorded only the twitch of the CTM in 15 cases (8.5%). In 20 cases, equal to 11.3%, we were unable to identify any signal from the EBSLN. In the 141 patients in which we recorded S1 signals, we identified an EBSLN attributable to Cernea type 1 in 61 cases (43.3%), Cernea type 2a in 54 cases (38.3%) and Cernea type 2b in 26 cases (18.4%) (Fig. 5).

**Fig. 6:**
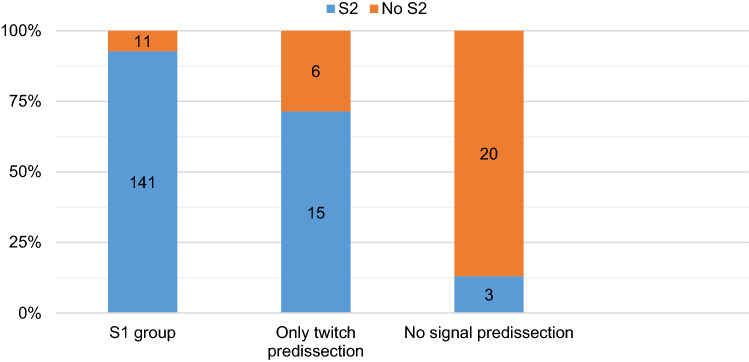
Absence of signal in three groups classified about the pre-dissection response. (S2 = signal positive; No S2 = Absence of signal). (*p* = 0.0001)

Upon analysis of the S2 results, we lost the S2 signal in 11/141 cases identified with IONM, equal to 7.8% of cases; in the cases in which S1 was identified only with twitch of the CTM, we lost the signal in 6/15 cases (40%). In total, adding the cases identified with IONM and muscle twitch, we lost the signal in 17 out of 156 nerves examined, equal to 10.9% of cases. In the 20 cases without S1 identification (we were unable to identify the external branch of the superior laryngeal nerve or muscle twitch), we confirmed the loss in 17 of 20 cases in the S2 evaluation, equal to 85%. The comparison between the two groups was statistically significant (*p* = 0.0001) (Fig. 6).

## Discussion

Surgical management of the EBSLN during thyroidectomy is complex, and in contrast to conventional RLN dissection, most clinicians tend to avoid, rather than consistently denude, identify and monitor, the EBSLN.

Optical recognition and identification of the EBSLN is challenging, particularly in patients with giant multinodular goitre, advanced thyroid cancer, remedial surgery, apical lesions or haemorrhage of the upper thyroid pole. The identification of the EBSLN can be easier in surgical procedures performed with video-assisted techniques, and even if it can lengthen the mean surgical time, it often improves surgical outcomes [10].

There is an increasing consideration of the potential role of EBSLN estimation, monitoring, functional preservation and evaluation in thyroid and parathyroid surgery. At the end of the surgical procedure, intraoperative EBSLN monitoring can provide postoperative neural function prognosis, particularly after EBSLN stimulation (performed in the most cranial EBSLN segment, i.e., cranially to the superior pole region surgically managed, namely, S2) A positive CTM twitch and EMG waveform are reliable evidence for functional EBSLN preservation. In the same way, S1 is an important surgical step for EBSLN identification. S1 is able to help the surgeon identify the EBSLN during the isolation of the upper thyroid pole by means of electromyographic signal mapping, avoiding the section of the vascular structures before EBSLN identification [11–13].

There are few studies in the literature that deal with the topic of EBSLN neuromonitoring, but according to this preliminary report, this S1 approach, which is safe and easily reproducible if the appropriate IONM devices are available, shows excellent results if consistently used and properly adopted.

In fact, our data clearly show that stimulation and recognition of EBSLN, performed before any dissection manoeuvre close to the superior vascular thyroid pole, leads to a much higher rate of nerve conservation, confirmed by IONM [14]. Furthermore, when the nerve is not clearly identified prior to dissection, the nerve preservation rate drops dramatically [15].

We also found that the pre-dissection EBSLN electromyographic signal was significantly lower than the post-dissection signal, when present, likely due to the compressive effect of the gland on the nerve itself.

The principal limits of our study are the small number of NARs examined and that we did not routinely perform vocal cord stroboscopy or postoperative transcutaneous laryngeal ultrasound in all patients.

The “*External Branch of the Superior Laryngeal Nerve Monitoring During Thyroid and Parathyroid Surgery: International Neuromonitoring Study Group Standards Guideline Statement*” was published by the INMSG in 2013 [4], with the aim of describing a standardized approach for EBSLN monitoring, improving the practice of EBSLN monitoring, and optimizing the clinical utility of the IONM technique. The INMSG proposed the EBSLN mnemonic formula and steps for safe identification, dissection, and preservation of the nerve:

(E) Exposing the space hosting the EBSLN. EBSLN exposure can be enhanced by transverse bisection of the laryngeal head muscle and slight pulling of the superior lobe in the lateral and caudal directions.

(B) Blunt dissection within the avascular space between the CTM and the medial aspect of the superior pole. This approach allows visual identification of the EBSLN located on the inferior constrictor muscle before its termination at the internal CTM.

(S) Continuously stimulating the tissues during EBSLN dissection. Stimulation of the tissues during blunt dissection should be undertaken to facilitate optical localization of the EBSLN.

(L) Continuous visualization for CTM contraction. The search for a positive CTM contraction is suggested during delicate dissection of tissues with the stimulation probe tip rather than expecting a positive EMG response on the monitor.

(N) Accompany anatomization using IONM mapping. When the EBSLN is not identified but mapped in the surgical area, this technique should maximize the division level of the superior vessels to secure the intact functional continuity of the EBSLN provided by electrical nerve tests. Medially positive stimulation is obtained, and then, only the tissue is divided into the dissection of the superior pole, which stimulates negatively, laterally [16].

In the literature, EBSLN lesions are reported in up to 58% of cases without identification [5]. A meta-analysis showed an incidence of temporary EBSLN injury of 1.4% with the use of IONM, which increased to 5.7% when the nerve was identified by visualization alone. Permanent injury was shown to occur in 0.3% of cases in the IONM group versus 0.9% of the vessels in the visualization group [17].

In conclusion, intraoperative neuromonitoring of the EBSLN appears to be a safe tool for identifying the nerve and confirming the preservation of its functionality.

## Data Availability

All patients’ data were collected in a prospective manner with a dedicated electronic Microsoft Office Access Data Base (Microsoft Corp, Redmond, Wash).
